# Outcome of Critically Ill Patients with Testicular Cancer

**DOI:** 10.1155/2017/3702605

**Published:** 2017-10-26

**Authors:** Silvio A. Ñamendys-Silva, Mireya Barragán-Dessavre, Andoreni R. Bautista-Ocampo, Francisco J. García-Guillén, Bertha M. Córdova-Sánchez, Edgar Constantino-Hérnandez, Paulina Correa-García, Octavio González-Chon, Angel Herrera-Gómez

**Affiliations:** ^1^Department of Critical Care Medicine, Instituto Nacional de Cancerología, 14080 Mexico City, Mexico; ^2^Department of Critical Care Medicine, Instituto Nacional de Ciencias Médicas y Nutrición Salvador Zubirán, 14000 Mexico City, Mexico; ^3^Department of Critical Care Medicine, Fundación Clínica Médica Sur, 14050 Mexico City, Mexico

## Abstract

**Purpose:**

To evaluate the clinical characteristics and outcomes of critically ill patients with testicular cancer (TC) admitted to an oncological intensive care unit (ICU).

**Methods:**

This was a prospective observational study. There were no interventions.

**Results:**

During the study period, 1,402 patients with TC were admitted to the Department of Oncology, and 60 patients (4.3%) were admitted to the ICU. The most common histologic type was nonseminomatous germ cell tumors (55/91.7%). The ICU, hospital, and 6-month mortality rates were 38.3%, 45%, and 63.3%, respectively. The Cox multivariate analysis identified the white blood cells count (HR = 1.06, 95% CI = 1.01–1.11, and *P* = 0.005), ionized calcium (iCa) level (HR = 1.23, 95% CI = 1.01–1.50, and *P* = 0.037), and 2 or more organ failures during the first 24 hours after ICU admission (HR = 3.86, 95% CI = 1.96–7.59, and *P* < 0.001) as independent predictors of death for up to 6 months.

**Conclusion:**

The ICU, hospital, and 6-month mortality rates were 38.3%, 45%, and 63.3%, respectively. The factors associated with an increased 6-month mortality rate were white blood cells count, iCa level, and 2 or more organ failures during the first 24 hours after ICU admission.

## 1. Introduction 

Testicular cancer (TC) represents between 1% and 1.5% of male neoplasms. TC shows excellent cure rates. Germ cell tumors (GCTs) are classified as seminoma or nonseminoma. More than 90% of patients diagnosed with GCTs are cured, including 70% to 80% with advanced tumors who are treated with chemotherapy [1]. The main factors contributing to this outcome are careful staging at the time of diagnosis; adequate early treatment based on chemotherapeutic combinations, with or without radiotherapy and surgery; and very strict follow-up and salvage therapies [2]. Critically ill patients with TC may require intensive care due to different causes such as acute respiratory failure within a few days of initiation of the chemotherapy [3], postanesthetic recovery, infection, and sepsis; however no studies have reported the prognosis of this group of critically ill patients who require intensive care. Because of this limitation, we decided to perform the present study, aiming to evaluate the clinical characteristics and outcomes of critically ill patients with TC admitted to an oncological intensive care unit (ICU).

## 2. Methods

This was a prospective observational study of 60 consecutive critically ill cancer patients with TC admitted to the ICU of the Instituto Nacional de Cancerología (INCan), located in Mexico City, from February 2008 to February 2015. This study was approved by the Bioethics Committee of INCan, and the requirement for informed consent was waived (Rev/09/15).

Demographic, clinical, and laboratory data were collected during the first day of the ICU stay and included the primary histology, sites of metastasis, International Germ Cell Consensus Classification [4], performance status (Eastern Cooperative Oncology Group scale) [5] during the last month before hospitalization, tumor markers, need for mechanical ventilation (MV), need for vasopressor therapy, need for hemodialysis, length of invasive MV, length of stay in the hospital wards before ICU admission, and outcome data (ICU, hospital, and 6-month mortality rate). The length of the ICU stay was measured as the number of days from ICU admission until ICU discharge or death. The length of stay in the hospital before ICU admission was measured as the number of days from admission to the hospital until ICU admission. The Acute Physiology and Chronic Health Evaluation II score [6] and the Mexican Sequential Organ Failure Assessment (MEXSOFA) score [7] were calculated using the worst values for acute physiological variables during the first 24 hours after admission to the ICU. Organ failure was defined as a MEXSOFA score >2 for any of the five organs/systems evaluated. The patients were divided into two groups: survivors and nonsurvivor.

### 2.1. Data Presentation and Statistical Analyses

For the statistical analyses we followed the methods of Ñamendys-Silva et al. [8]. Continuous variables are expressed as the means ± standard deviation or as medians and interquartile ranges (IQRs), if the distribution was skewed. Categorical variables are expressed as the percentage. Student's *t*-test or the Mann–Whitney *U*-test were used to compare continuous variables according to the data distribution (normal or nonnormal, resp., determined using the Kolmogorov–Smirnov test), and the chi squared or Fisher's exact test was used to compare categorical variables.

Cox proportional hazards univariate and multivariate analysis were used to identify factors with potential prognostic significance for 6-month survival. Variables selected in the univariate analysis (*P* < 0.25) and those considered clinically relevant were included in a multivariable Cox proportional hazards regression model to estimate the independent contribution of each variable to the mortality. The results were reported using hazard ratios (HRs) and the corresponding 95% confidence intervals (95% CIs). Survival time was defined as the time (days) from ICU admission to death from any cause up to 6 months of follow-up. Patient survival was analysed using the Kaplan–Meier method and compared between groups by the log-rank test. A two-sided *P* value < 0.05 was used to determine statistical significance. Statistical analyses were performed using the Statistical Package for the Social Sciences software (version 22.0; SPSS, Chicago, IL, USA).

## 3. Results

**Figure 1:**
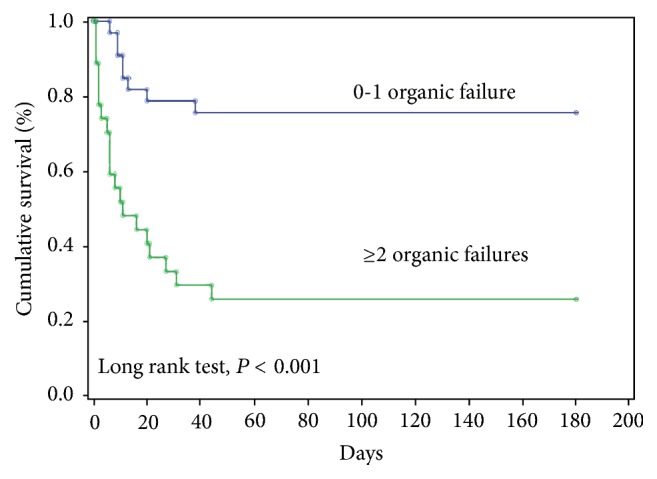
Six-month survival by number of organ failures.

During the study period, 1,402 patients with TC were admitted to the Department of Oncology of the INCan, and 60 patients (4.3%) were evaluated by intensivists at the request of ward oncologists responsible for the patient. The 60 patients with TC were admitted to the ICU of the INCan. The mean age of the patients was 28.3 ± 8.2 years. The most common histologic type was nonseminomatous germ cell tumors (55/91.7%), and 5 (8.3%) were seminomas. In total 98.3% of the patients underwent an orchiectomy. After the orchiectomy 14 patients (23.3%) received radiotherapy, and 52 patients (86.7%) received chemotherapy. Sixty percent of the patients received first-line therapy. Those patients who experienced an incomplete response to first-line therapy were treated with second-line therapy (16/30.7%). Forty-nine patients (81.7%) were classified as having a poor prognosis, 9 patients (15%) as having an intermediate prognosis, and 2 patients (3.3%) as having a good prognosis. The main reasons for ICU admission were septic shock and postoperative care in 17 patients (28.3%), respectively, followed by acute respiratory failure in 13 patients (21.7%). The incidence of organ failure was noted most frequently for the respiratory (33/55%), cardiovascular (26/43.3%), and renal systems (12/20%). During the first 24 hours of ICU admission, invasive MV was required by 43 patients (71.6%) for a median duration of 3 (interquartile range, 1–7) days, and vasopressors were required for 27 patients (24%). The median length of stay in the hospital wards before ICU admission was 2 days (1–7) (Table 1). The length of stay in the ICU and hospital was 3 (2–6) and 8 (5–15) days, respectively.  Table 2 lists the main characteristics of the patients according to the presence of hospital mortality and Table 3 presents the laboratory parameters according to hospital mortality. The ICU, hospital, and 6-month mortality rates were 38.3%, 45%, and 63.3%, respectively. The Cox multivariate analysis identified the white blood cells count (HR = 1.06, 95% CI = 1.01–1.11, and *P* = 0.005), ionized calcium (iCa) level (HR = 1.23, 95% CI = 1.01–1.50, and *P* = 0.037), and 2 or more organ failures during the first 24 hours after ICU admission (HR = 3.86, 95% CI = 1.96–7.59, and *P* < 0.001) as independent predictors of death for up to 6 months (Table 4). The six-month survival by number of organ failures is presented in Figure 1.

## 4. Discussion 

In this study of 60 critically ill patients with TC who were admitted to the ICU, the ICU, hospital, and 6-month mortality rates were 38.3%, 45%, and 63.3%, respectively. To the best of our knowledge, no other group has conducted any study in these patients. Patients who had two or more organ system failures during the first 24 hours after ICU admission had a high mortality rate. Germ cell tumors (GCTs) are uncommon tumors that constitute only 2% of all human malignancies, but they are the most common solid tumor in men between 15 and 34 years of age [1]. GCTs are highly sensitive to radiotherapy and chemotherapy; however, stage is an important predictor of survival and patients with poor prognosis have a five-year survival rate of approximately 50% [9]. In this study, more than 80% of patients were in advanced stages of the disease at ICU admission; this could be related to a low survival (37%) to 6 months.

In our series, the independent predictors of death at 6 months were white blood cells count, ionized calcium level, and 2 or more organ failures during the first 24 hours after ICU admission.

An elevated white blood cell count typically reflects the normal response of bone marrow to an infectious or inflammatory process [10, 11]. A high white blood cell count is associated with an increased risk of cancer-related mortality. Local inflammatory processes that have long been known to be associated with tumor progression may be reflected in the systemic inflammatory marker of a higher white blood cell count [12]. Approximately one-third of the patients included in our study were admitted to the ICU with septic shock, and more than 80% were in advanced stages of the disease; both scenarios are possibly related to the fact that leukocytosis is a factor independent of death in critically ill patients with TC. Although the serum tumor markers alpha-fetoprotein, lactate dehydrogenase, and *β*-human chorionic gonadotropin (*β*-HCG) are critical in diagnosing GCTs, determining the prognosis, and assessing the treatment outcome [1], these biomarkers were not predictors of a poor prognosis in patients with testicular cancer during their stay in the ICU. The serum levels of these biomarkers should not be considered when deciding whether a patient is eligible to be admitted to the ICU. The white blood cell count is advantageous due to its simplicity, low cost, and availability and may be used to identify patients at high risk of poor outcomes.

In hospitalized cancer patients the reported incidence of hypocalcaemia is less than 11% [13]. The measurement of serum iCa is common in patients admitted to ICUs, at least half of which will have values outside the reference range during the ICU stay. The majority of these patients do not have an underlying disease of calcium homeostasis [14]. Derangements in iCa levels occur very commonly in critically ill patients, especially those with sepsis, and are usually not the result of an underlying disease of calcium homeostasis [14]. In nonseptic, critically ill adults, hypocalcaemia has also been reported [15]. Hypocalcaemia as a prognostic factor in critically ill cancer patients has never been studied. In our series, the iCa levels were low in both survivors and nonsurvivors during their hospital stay; however, ionized calcium level was an independent factor of death at six months. Measures of iCa during the ICU and hospital stay may prevent asymptomatic stages of hypocalcaemia or lead to their early detection and correction, as well as improve the outcomes of critically ill patients with TC.

The main prognostic factors in critically ill cancer patients admitted to the ICU are degree of dysfunction, number of organ failures, and performance status at ICU admission [16–18]. The results suggest that the number of organ failures during the ICU stay of patients with TC appears crucial to predicting the outcome. Consequently, organ failure may be a simple and objective tool for oncologists and intensivists to identify patients who should be admitted earlier to the ICU.

Our study has some limitations in that it was undertaken at a single center, which may restrict the generalizability, and the sample size was small.

## 5. Conclusion

The ICU, hospital, and 6-month mortality rates were 38.3%, 45%, and 63.3%, respectively. The factors associated with an increased 6-month mortality rate were white blood cells count, iCa level, and 2 or more organ failures during the first 24 hours after ICU admission.

## Figures and Tables

**Table 1:** Clinical characteristics of critically ill patients with testicular cancer admitted to the intensive care unit (*n* = 60).

Characteristic	Finding
*Age, years, mean ± SD*	28.3 ± 8.2
*Primary histology, n (%)*	
Nonseminomatous germ cell tumors	55 (91.7)
Seminoma	5 (8.3)
Metastasis	58 (96.7)
*Sites of metastasis, n (%)*	
Pulmonary	6 (10)
Extrapulmonary	17 (28.3)
Mixed	35 (58.3)
*Stages, n (%)*	
IIB/IIC	8 (13.3)
IIIA/IIIB/IIIC	52 (86.7)
*IGCCCG, n (%)*	
Good	2 (3.3)
Intermediate	9 (15.0)
Poor	49 (81.7)
*Tumor marker, median (IQR)*	
*α*-Fetoprotein, *µ*g/L	4.17 (2.11–173)
Human chorionic gonadotropin, U/L	2.02 (0–12,189)
Lactate dehydrogenase, U/L	421.5 (194.5–1,304.5)
*Characteristics on admission to the ICU*	
Need for vasopressors, *n* (%)	27 (45)
Need for IMV, *n* (%)	43 (41.7)
Need for hemodialysis, *n* (%)	1 (1.7)
Length of IMV (days), median (IQR)	3 (1–7)
PEEP, median (IQR)	6 (5–9)
In hospital wards time before ICU admission, *n* (%)	49 (81.6)
Length of stay in hospital wards before ICU admission, (days), median (IQR)	2 (1–7)
APACHE score, mean ± SD	14.7 ± 6.4
MEXSOFA, mean ± SD	6.7 ± 4.0
*Performance status, n (%)*	
0–2	34 (56.7)
3-4	26 (43.3)
*Outcome data*	
Length of stay in ICU, days, median (IQR)	3 (2–6)
Length of hospital stay, days, median (IQR)	8 (5–15)
ICU mortality, *n* (%)	23 (38.3)
Hospital mortality, *n* (%)	27 (45.0)
6-month mortality, *n* (%)	38 (63.3)

SD, standard deviation. IMV, invasive mechanical ventilation. IQR, interquartile range. ICU, intensive care unit. SOFA, sequential organ failure assessment. APACHE, Acute Physiology and Chronic Health Evaluation. SAPS, Simplified Acute Physiology Score. IGCCCG, the International Germ Cell Cancer Collaborative Group.

**Table 2:** Characteristics of the patients according to the presence of hospital mortality.

Variable	Survivor (*n* = 32)	Nonsurvivor (*n* = 28)	*P*
*Age, years, mean ± SD*	27.2 ± 8.4	29.5 ± 7.8	0.271
*APACHE II, point, mean ± SD*	13 ± 6.39	16.6 ± 6	0.033
*MEXSOFA, mean ± SD*	4.7 ± 3.3	8.9 ± 3.6	<0.001
*Primary histology, n (%)*			
Nonseminomatous germ cell tumors	30 (93.7)	25 (89.2)	0.657
Seminomatous germ cell tumors	2 (6.2)	3 (10.7)	
*Metastasis, n (%)*			
No	0 (0)	2 (7.1)	0.214
Yes	32 (100)	26 (92.8)	
*Sites of metastasis, n (%)*			
Pulmonary	2 (6.2)	4 (15.3)	0.408
Extrapulmonary	11 (34.3)	6 (23)	
Mixed	19 (59.3)	16 (61.5)	
*IGCCCG risk classification*			
Good	0 (0)	2 (7.1)	0.240
Intermediate	4 (12.5)	5 (17.8)	
Poor	28 (87.5)	21 (75)	
*ECOG performance status, n (%)*			
0–2	20 (62.5)	14 (50)	0.330
3-4	12 (37.5)	14 (50)	
*Tumor Marker, median (IQR)*			
*α*-fetoprotein, *µ*g/L	3.53 (2.19–27)	7.28 (2.0–336.5)	0.662
Human chorionic gonadotropin, U/L	0 (0–2.98)	12,189 (13–191,015)	<0.001
Lactate dehydrogenase, U/L	214 (155.5–361.5)	1,251 (465.5–2,361.5)	<0.001
*Need for vasopressors, n (%)*			
Yes	24 (75)	9 (32.1)	0.001
No	8 (25)	19 (67.8)	
*Need for IMV, n (%)*			
Yes	19 (59.3)	24 (85.7)	0.024
No	13 (40.7)	4 (14.3)	
*Length of IMV, day*	1 (0–4)	2.5 (1–8.5)	0.015
*Length of stay in ICU, median, (IQR)*	2.5 (2–5)	3 (1.5–8.5)	0.001
*Length of stay in hospital wards before ICU admission, median (IQR)*	1.5 (1–5)	1 (0–6)	<0.001

APACHE, Acute Physiology and Chronic Health Evaluation. ICU, intensive care unit. IGCCCG, International Germ Cell Cancer Collaborative Group. ECOG, Eastern Cooperative Oncology Group. IMV, invasive mechanical ventilation.

**Table 3:** Laboratory parameters according to hospital mortality.

Variable	All patients (*n* = 60)	Survivor (*n* = 32)	Nonsurvivor (*n* = 28)	*P*
*Hemoglobin, g/dl, mean ± SD *	10.1 ± 2.3	10.9 ± 1.9	9.1 ± 2.3	0.001
*White blood cells count*, 10^3^ cells/mm^3^	12.4 ± 7.4	10.7 ± 4.6	14.4 ± 9.5	0.065
Neutrophils, 10^3^ cells/mm^3^	10.5 (6.15–14.5)	9.45 (5.3–11.7)	11.25 (7.8–16.9)	0.087
Lymphocytes, 10^3^ cells/mm^3^	0.7 (0.4–1.2)	0.75 (0.5–1.0)	0.55 (0.3–1.6)	0.672
Platelets, 10^3^ cells/mm^3^	187.5	196.2 ± 121.2	177.7 ± 117.7	0.552
*Serum creatinine*, mg/dL	0.9 (0.7–1.6)	0.88 (0.7–1.3)	1.1 (0.7–1.8)	0.310
*Glucose*, mg/dL	137 ± 44.3	135.5 ± 43.4	140.4 ± 46	0.672
*Uric acid*, mg/dL	5.7 ± 3	5.3 ± 2.5	6.2 ± 3.5	0.276
*Serum electrolytes *				
Sodium, mEq/L	136.7 ± 4.2	136.5 ± 4.1	137.1 ± 4.4	0.588
Potassium, mEq/dL	4.4 ± 0.9	4.1 ± 0.6	4.7 ± 1.2	0.029
Chlorine, mEq/dL	106.9 ± 6.3	106.9 ± 7	106.9 ± 5.7	0.998
Magnesium, mg/dL	2 ± 0.4	1.9 ± 0.4	2.2 ± 0.4	0.025
Phosphorus, mg/dL	4.2 (3.6–5.2)	4.1 (3.1–4.6)	4.4 (3.7–6.2)	0.073
Ionized calcium, mmol/L	0.8 (0.7–1)	0.8 (0.7–1)	0.8 (0.8–1)	0.794
*Total bilirubin*, mg/dL	1.1 (0.7–2.1)	0.8 (0.6–1.8)	1.5 (0.9–2.9)	0.013
*Albumin*, g/dL	2.1 (1.6–2.8)	2.5 (1.9–3.1)	1.8 (1.5–2.4)	0.002
*Lactate*, mmol/L				
ICU admission	2 (1.4–3.5)	2.3 (1.4–3.4)	1.7 (1.5–3.6)	0.772
Stay in ICU	1.5 (1.1–2)	1.4 (0.9–1.9)	1.9 (1.3–2)	0.038

ICU, intensive care unit.

**Table 4:** Univariable and multivariable analysis of factors associated with 6-month mortality (*n* = 60).

Variable	Hazard ratio	95% CI	*P*	Hazard ratio	95% CI	*P*
	Univariate			Multivariate		
*APACHE II*	1.04	0.99–1.09	0.570			
*MEXSOFA*	1.12	1.04–1.21	0.001			
*Hemoglobin,* gr/dL	0.79	0.68–0.93	0.005			
*White blood cells*	1.05	1.005–1.10	0.032	1.06	1.01–1.11	0.005
Lymphocytes, 10^3^ cells/mm^3^	0.99	0.86–1.13	0.911			
Platelets, 10^3^ cells/mm^3^	1.00	0.99–1.00	0.845			
Neutrophils, 10^3^ cells/mm^3^	1.05	0.99–1.10	0.590			
*Serum creatinine*, mg/dL	1.00	0.92–1.09	0.915			
*Glucose*, mg/dL	0.99	0.99–1.00	0.863			
*Uric acid*, mg/dL	1.06	0.95–1.18	0.245			
*Serum electrolytes*						
Sodium, mEq/L	1.01	0.94–1.10	0.661			
Potassium, mEq/L	1.54	1.10–2.14	0.011			
Chlorine, mEq/L	0.98	0.93–1.03	0.500			
Magnesium, mg/dL	2.25	1.11–4.57	0.024			
Phosphorus, mg/dL	1.25	1.09–1.45	0.002			
Ionized calcium, mmol/L	1.17	0.96–1.43	0.103	1.23	1.01–1.50	0.037
*Total bilirubin*, mg/dL	1.06	0.94–1.19	0.284			
*Albumin*, g/dL	0.88	0.69–1.14	0.356			
*Lactate*, mmol/L						
ICU admission	0.92	0.77–1.10	0.361			
Stay in ICU	1.28	0.91–1.80	0.146			
*ECOG, (3-4)*	1.53	0.80–2.90	0.190			
*≥2 organic failures*	3.09	1.61–5.95	0.001	3.86	1.96–7.59	<0.001

ICU, intensive care unit. APACHE, Acute Physiology and Chronic Health Evaluation. ECOG, Eastern Cooperative Oncology Group.
